# Einsatz von Nahtverschlusssystemen in der interventionellen Rhythmologie: Einfluss auf Abläufe, Ressourcenverbrauch, Vergütung und Patientensicherheit im Krankenhaus im Rahmen der STYLE-AF-Studie

**DOI:** 10.1007/s00399-025-01095-5

**Published:** 2025-07-22

**Authors:** Sorin Ștefan Popescu, Christian Elsner, Noemi Kucharz, Valerie Zu Rhein, Clarissa Engewald, Kai Pardey, Roland Richard Tilz

**Affiliations:** 1 Klinik für Rhythmologie, Universitätsklinikum Schleswig-Holstein, Campus Lübeck, Lübeck, Deutschland; 2 Standort Hamburg/Kiel/Lübeck, Deutsches Zentrum für Herz-Kreislauf-Forschung, Lübeck, Deutschland; 3 Zentrum für künstliche Intelligenz, Universität zu Lübeck, Ratzeburger Allee 160, 23562 Lübeck, Deutschland; 4 PwC Deutschland, Am Alsterufer 1, 20354 Hamburg, Deutschland; 5 Kaufmännische Direktion, Universitätsklinikum Schleswig-Holstein, Campus Lübeck Ratzeburger Allee 160, 23562 Lübeck, Deutschland

**Keywords:** Tagesstationäre Behandlung, Prozess Management, Gefäßverschluss System, Gesundheitsökonomie, Aufwachraum Management, Day care treatment, Process Management, Vessel Closure, Post-Anesthesia Care Management, Health Economics

## Abstract

**Hintergrund und Ziele:**

Die Katheterablation kardialer Arrhythmien erfolgt meist über einen venösen Leistenzugang. Zur Vermeidung von Blutungen und Verkürzung der Liegedauer gewinnen venöse Nahtverschlusssysteme an Bedeutung. Ziel war die quantitative Bewertung des Einflusses von Nahtverschlusssystemen, tagesstationärer Abrechnung und früher Entlassung aus dem Aufwachraum (AWR) auf ökonomische Kennzahlen, patientenbezogene Endpunkte und Personalauslastung.

**Methoden:**

Basierend auf Daten der STYLE-AF-Studie wie Verweildauer im AWR, Blutungen in der Leiste und weiteren Outcomeparametern, ergänzt durch Experteninterviews und Literatur, wurden mittels Simulationsstudie und realer Implementierung Szenarien analysiert: Einsatz von Nahtverschlusssystemen, tagesstationäre Abrechnung, frühe AWR-Entlassung und deren Kombinationen. Primäre Endpunkte waren die Zeitersparnis im AWR sowie die Veränderung der Leistenblutungswahrscheinlichkeit, respektive Patientenkomfortgewinne über QALYs.

**Ergebnisse:**

Die alleinige Nutzung von Nahtverschlusssystemen führte zu 6,5 % mehr Fällen und einem Zugewinn von 0,0034 Quality-Adjusted Life Years (QALYs) pro Patient:in. Kombiniert mit tagesstationärer Abrechnung stieg der Deckungsbeitrag pro Fall um 415 €. Ein Wechsel zur tagesstationären Abrechnung ohne Nahtverschlusssysteme ergab 388,80 € Mehrertrag, aber keinen QALY-Gewinn oder Personalentlastung. Die größte Wirkung zeigte die Kombination aller Maßnahmen: 25 % höhere Fallzahlen, Deckungsbeitragssteigerung von 661,27 € pro Patient:in, bis zu 24 % relative Personalentlastung im AWR und einen QALY-Zuwachs von 0,0034.

**Schlussfolgerung:**

Nahtverschlusssysteme bringen, insbesondere in Kombination mit Prozessanpassungen und tagesstationärer Abrechnung, erhebliche multidimensionale Vorteile für Wirtschaftlichkeit (Deckungsbeitrag), Ressourceneffizienz (Zeit im AWR) und Patientenoutcomes (QALYs über reduzierte Leistenblutungen) – abhängig von klinikindividuellen Rahmenbedingungen und konsequenter Umsetzung.

## Abstract (en)

**Background and objectives:**

Catheter ablation of cardiac arrhythmias is typically performed via femoral venous access. To reduce bleeding complications and shorten hospital stays, venous closure devices are gaining importance. This study aimed to quantitatively evaluate the impact of closure devices, “tagesstationär” (2 day-case stay with night leave) billing, and early recovery room (post-anesthesia care unit, PACU) discharge on economic indicators, patient-related outcomes, and staffing requirements.

**Methods:**

Based on data from the STYLE-AF study—including PACU length of stay, groin bleeding rates, and additional outcome parameters—supplemented by expert interviews and literature review, we conducted a simulation study and real-world implementation to analyze various scenarios: use of closure devices, “tagesstationär” discharge billing, early PACU discharge, and combinations thereof. Primary endpoints were time savings in the PACU, changes in groin bleeding probability, and gains in patient comfort quantified by quality-adjusted life years (QALYs).

**Results:**

The exclusive use of closure devices led to a 6.5% increase in case throughput and a gain of 0.0034 QALYs per patient. When combined with same-day discharge billing, the contribution margin increased by € 415 per case. Switching to same-day discharge billing without closure devices yielded a € 388.80 higher revenue per case but did not result in QALY gains or staff workload reductions. The most substantial effects were observed with the combined implementation of all measures: a 25% increase in case volume, € 661.27 higher contribution margin per patient, up to 24% relative reduction in PACU staff workload, and a QALY gain of 0.0034.

**Conclusion:**

Closure devices, particularly when integrated with process optimizations and “tagesstationär” discharge billing, provide significant multidimensional benefits across economic performance (contribution margin), resource efficiency (PACU time), and patient outcomes (QALYs through reduced groin bleeding)—contingent on institutional context and consistent implementation.

Kardiologische Interventionen über einen venösen Zugang, darunter Katheterablationen und der Verschluss des linken Vorhofohrs („left atrial appendage occlusion“, LAAO), sind etablierte Verfahren zur Behandlung von Herzrhythmusstörungen und zur Schlaganfallprävention. Die Zahl dieser Eingriffe nimmt kontinuierlich zu. Laut dem Deutschen Herzbericht 2024 [1] lag die altersstandardisierte Hospitalisationsrate für Herzrhythmusstörungen im Jahr 2022 bei 485,7 pro 100.000 Einwohner:innen. Mit der demografisch bedingten Zunahme kardiovaskulärer Erkrankungen wird in den kommenden Jahrzehnten eine deutliche Steigerung der Inanspruchnahme invasiver kardiologischer Therapien erwartet [2]. Der transfemorale Zugang stellt den Standardweg für die Mehrzahl dieser Prozeduren dar [3]. Die Hämostase erfolgt nach wie vor durch manuelle Kompression, mit oder ohne Anwendung einer Z‑Naht, gefolgt von der Anlage eines Kompressionsverbands in der Leiste, der für mehrere Stunden belassen wird – ein Verfahren, das eine verlängerte Überwachung im Aufwachraum (AWR) sowie auf Normalstationen erforderlich machen kann [4].

Um vaskuläre Komplikationen zu reduzieren und gleichzeitig Behandlungsdauer und Ressourceneinsatz zu optimieren, kommen zunehmend Nahtverschlusssysteme zum Einsatz [5, 6]. Parallel dazu gewinnen prozessuale Anpassungen an Relevanz – insbesondere die Umstellung von vollstationären auf tagesstationäre Abrechnungsmodi sowie die frühzeitige Entlassung aus dem AWR nach Erreichen der Mobilisierungszeit, definiert als die durchschnittliche Zeit bis zur sicheren Mobilisierung der Patient:innen. Ziel der hier dargestellten Studie war es, den realen und praktischen Einfluss der Nutzung von Nahtverschlusssystemen in Kombination mit weiteren Maßnahmen auf ökonomische Kennzahlen, patientenbezogene Endpunkte und Personalauslastung zu untersuchen und zu quantifizieren.

## Methodik

### Studiendesign

Um die Auswirkungen des Einsatzes von Nahtverschlusssystemen im klinischen Alltag systematisch zu untersuchen, wurde eine Simulationsstudie auf Basis eines datenbasierten Modells durchgeführt. Begleitend erfolgte eine Beobachtung der praktisch relevanten Effekte im Krankenhausbetrieb nach Systemeinführung. Die Datengrundlage des Modells stützt sich auf drei komplementäre Quellen: Primärdaten aus der STYLE-AF-Studie [6], Beobachtungen entlang des Patientenpfads sowie qualitative Experteninterviews mit Kardiolog:innen und Pflegefachkräften und auf wissenschaftliche Sekundärdaten, welche übergreifende Informationen zu Kostenstrukturen, Komplikationsraten, Liegezeiten und Abrechnungsmechanismen bereitstellen.

Die STYLE-AF-Studie war eine prospektive, multizentrische, randomisierte, kontrollierte Studie, die zwischen November 2022 und Januar 2024 an drei deutschen Zentren durchgeführt wurde. Eingeschlossen wurden Patient:innen, ab einem Alter von 18 Jahren, die sich einer elektiven Katheterablation bei Vorhofflimmern unter Verwendung einer Einführschleuse und maximal zwei femoralen Venenzugängen unterzogen. Ausschlusskriterien umfassten u. a. aktive systemische oder kutane Infektionen, weitere medizinische Parameter sowie prozedurale Komplikationen, die den regulären postinterventionellen Verlauf, die Mobilisierung oder die Entlassungszeit beeinträchtigten, eine fehlerhafte Schleusenplatzierung (z. B. arterielle Fehlplatzierung), intraprozedurale Blutungs- oder Thromboseereignisse sowie zugangsbezogene Kriterien, die einen problemfreien Gefäßzugang oder die korrekte Lage der Schleuse verhinderten. Die Patient:innen wurden im Verhältnis 1:1 randomisiert und erhielten eine Hämostase entweder mittels eines venösen Nahtverschlusssystems oder mit einer Z‑Naht in Kombination mit manueller Kompression. Der primäre Wirksamkeitsendpunkt war die Zeit bis zur Mobilisierung, während der primäre Sicherheitsendpunkt die Inzidenz schwerwiegender periinterventioneller Komplikationen bis zur Entlassung aus dem Krankenhaus darstellte. Sekundäre Endpunkte umfassten das Auftreten von Komplikationen innerhalb der ersten 30 Tage nach der Ablation, die Zeit bis zur Hämostase, die Zeit bis zur Erfüllung der Entlassungskriterien, den tatsächlichen Entlassungszeitpunkt sowie das subjektive Komfort- und Schmerzempfinden der Patientinnen und Patienten [6].

Methodisch basierte das Modell auf einer klassischen gesundheitsökonomischen Kosten-Nutzen-Rechnung mit Endpunkten der Wirtschaftlichkeit (Deckungsbeitrag), der Mitarbeiterentlastung (Reduktion Personalbindung und Patientenverweildauer im AWR) sowie Steigerung des Patientenkomforts (Erzeugung von QALYs durch Reduktion der Leistenblutung).

### Datenerhebung und -aufbereitung

#### Primärdaten

Die *STYLE-AF-Studie *[6] war weltweit die erste prospektive, randomisierte Studie, die die Sicherheit und Effizienz nahtbasierter Gefäßverschlusssysteme (Perclose™ ProGlide™, Abbott Vascular Deutschland GmbH, Wetzlar, Deutschland) im Vergleich zur herkömmlichen Z‑Naht mit manueller Kompression nach einer Pulmonalvenenisolation untersuchte. Die Ergebnisse zeigten in der Interventionsgruppe eine signifikant kürzere Zeit bis zur Blutstillung, Mobilisierung und Entlassungsfähigkeit. Zudem wurde eine geringere Inzidenz von milden vaskulären Komplikationen beobachtet. Zur Modellierung der Abläufe wurden diese Studiendaten für die Berechnung der Zeit bis zur Mobilisierung und Entlassung herangezogen (Tab. 1).

**Tab. 1:** Eingabeparameter Simulationsmodell

Parameter	Wert
Ziel-DRG	F50A
Anzahl Fälle pro Jahr	*n* = 1
Fall-Anwendungsquote Nahtverschlusssysteme	50 %
Nahtverschlusssystem pro Fall	1
Bundesbasisfallwert (2025)	4400,00 €
Neuer Erlös Ziel-DRG	6102,80 €
Abrechnungsart	Tagesstationär
Kosten pro Nahtverschlusssystem (Annahme)	Wert ist den Autoren bekannt und wird hier nicht veröffentlicht
Deckungsbeitrag Grenzkosten DRG	17,52 %
Opportunitätskosten pro Pflegetag	564,80 €
Entlassungsfähigkeit aus AWR in Minuten ohne Nahtverschlusssystem	340 min
Mobilisierungsfähigkeit aus AWR in Minuten ohne Nahtverschlusssystem	269 min
Entlassungsfähigkeit aus AWR in Minuten mit Nahtverschlusssystem	270 min
Mobilisierungsfähigkeit aus AWR in Minuten mit Nahtverschlusssystem	109 min
Blutungsevent-Rate ohne Nahtverschlusssystem	24 %
Blutungsevent-Rate mit Nahtverschlusssystem	9,5 %
Rate schwerer Leistenblutungen in typischer Patientenpopulation nach Kathetereingriff	2,3 %
QALY-Reduktion bei schwerer Leistenblutung bei Ereigniseintritt	0,23

Um ein tiefergehendes Verständnis des Patientenpfads zu erlangen, wurden die Prozessmodellierungen im Universitätsklinikum Schleswig-Holstein (UKSH) beobachtet und durch Experteninterviews abgesichert. Ein Beobachtungsprotokoll wurde erstellt, um die relevanten Aspekte systematisch zu erfassen und zu berichten [7]. Die Interviews waren semistrukturiert und lieferten ergänzende Informationen zu internen Prozessen, Abrechnungsart (tagesstationäre Abrechnung der DRG F50A^1^), der Anwendungsquote und den Kosten der Nahtverschlusssysteme, die in das Modell integriert wurden (Tab. 1).

#### Sekundärdaten zur Cross-Validierung

##### Abrechnungsdaten.

Gesundheitsökonomische Analysen belegen, dass die Kombination aus kürzerer Mobilisierungszeit durch Einsatz eines Nahtverschlusssystems [8] und reduzierter Blutungsneigung [9, 10] eine ökonomisch und patientenorientiert vorteilhafte DRG-Abrechnung ermöglicht, u. a. durch eine geringere Wiederkehrerquote. Dies schafft optimale Bedingungen für eine tagesstationäre Behandlung gemäß § 115e SGB V, bei der eine Entlassung am selben Tag medizinisch sicher erfolgen kann. Zur wirtschaftlichen Bewertung des Einsatzes von Nahtverschlusssystemen wurden die aG-DRG-Abrechnungsdaten des InEK, Stand 2024 [11] sowie die E1-Daten des UKSH herangezogen. Die Kalkulation erfolgt modellhaft auf Ebene eines einzelnen Eingriffs unter Verwendung des Bundesbasisfallwerts. Für eine einrichtungsspezifische Bewertung können lokale Fallzahlen und der jeweilige Landesbasisfallwert verwendet werden. Zur Abbildung der ökonomischen Effekte einer Umstellung auf die tagesstationäre Abrechnung wird bei der DRG F50A ein Abschlag gemäß der unteren Grenzverweildauer (ugVWD; [11]) angewandt. Zusätzlich erfolgt ein Abzug von 0,04 Relativgewichtspunkten gemäß § 115e SGB V. Ergänzend wird die Anwendungsquote von Nahtverschlusssystemen bezogen auf die Fallzahl sowie die Anzahl der verwendeten Nahtverschlusssysteme je Fall betrachtet. Dabei können auch Mehrfachpunktionen berücksichtigt werden. Basierend auf den Daten des UKSH wurde in der Modellrechnung der Standardfall mit einem Nahtverschlusssystem je Eingriff und einer durchschnittlichen Anwendungsquote von 50 % angenommen (Tab. 1).

##### Kosten.

Neben den Kosten für das Nahtverschlusssystem selbst (Tab. 1, dort aus wettbewerblichen Gründen nicht abgebildet, aber den Autoren bekannt) fließen auch die Abschläge der tagesstationären Behandlung gegenüber der ursprünglichen DRG-Vergütung in die Berechnungen ein. Demgegenüber steht der Wegfall stationärer Pflege- bzw. Übernachtungskosten, die als Opportunitäten im Deckungsbeitrag berücksichtigt werden. Der Deckungsbeitrag lässt sich aus den InEK-Daten [12] mit 17,52 % entnehmen (Tab. 1). Dazu wurde die gängige Methode angewendet, die Spalten der medizinischen und nichtmedizinischen Infrastrukturkosten der INeK-Systematik als Grenzkosten zu betrachten. Um die Opportunitätskosten für gewonnene Pflegetage auf der Station zu beziffern, wurden folgende Annahmen getroffen: Laut dem Statistischen Bundesamt beliefen sich die durchschnittlichen Kosten je stationärem Behandlungsfall im Jahr 2019 auf 5088 €, bei einer durchschnittlichen Verweildauer von 7,2 Tagen [13]. Daraus ergibt sich ein durchschnittlicher Umsatz pro Bett und Tag von rund 706 €. Die tatsächliche Bettenauslastung wird hierbei nicht gesondert berücksichtigt, da Betten als Engpassressource gelten und unterstellt wird, dass jeder nichtbelegte Tag mit einem alternativen Fall hätte genutzt werden können [14]. Diese Annahme scheint auch deswegen opportun, da zurecht davon ausgegangen werden kann, dass Krankenhäuser freiwerdende Ressourcen optimiert auslasten werden – mindestens zu Durchschnittsumsätzen, wahrscheinlich sogar darüber. Zur Berechnung dieser Opportunitätskosten müssen dann aber die variablen Kosten eines zusätzlichen Falls abgezogen werden. Diese können mit 20 % angesetzt werden, orientiert an einer bettenbezogenen Sachkostenquote im Krankenhaus [15]. Entsprechend ergibt sich ein Fixkostenanteil von 80 % (f = 0,8). Die Opportunitätskosten pro Patient:in lassen sich daher wie folgt berechnen: Opportunitätskosten = 706 € × 0,8 = 564,80 € (Tab. 1).

##### QALYs im Kontext von Leistenblutungen.

Resnic et al. [9] analysierten, dass der Einsatz von Nahtverschlusssystemen nicht nur kosteneffektiv ist, sondern auch zur Verbesserung der Lebensqualität der Patient:innen beitragen. Als entscheidenden patientenbezogenen Endpunkt hoben Gutzwiller et al. [10] die Bedeutung von QALYs in der Nutzenbewertung medizinischer Maßnahmen hervor. Studien [9, 16, 17] zeigen, dass schwere Blutungskomplikationen im Leistenbereich mit einem QALY-Verlust von etwa 0,23 pro betroffene Person einhergehen. Basierend auf den Daten der STYLE-AF-Studie [6], in der bei Anwendung eines Nahtverschlusssystems eine signifikant niedrigere Blutungsrate von 9,5 % im Vergleich zu 24,2 % bei konventioneller Hämostase festgestellt wurde und unter der Annahme, dieses Verhältnis ist auf schwere Blutungsereignisse projizierbar, lässt sich ein relevanter Effekt auf die gesundheitsbezogene Lebensqualität ableiten (Tab. 1).

### Modellaufbau

**Abb. 1:**
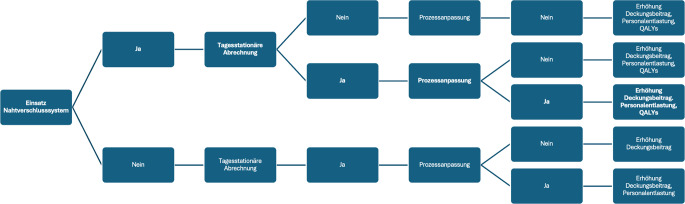
Entscheidungsbaum zur Analyse potenzieller Effekte eines Nahtverschlusssystems

Die Ergebnisse der STYLE-AF-Studie [6], die Beobachtungen und Interviews sowie die Literaturrecherche wurden systematisch in das ökonomische Modell integriert. Auf dieser Datengrundlage wurden über einen Entscheidungsbaum (Abb. 1) die Zielparameter des Modells definiert. Hieraus ergeben sich 5 Szenarien, deren Auswirkungen im Modell berechnet werden: Als Ausgangspunkt dient die Entscheidung über den Einsatz eines Nahtverschlusssystems. Wird dieses eingesetzt, folgt die Prüfung, welche Auswirkungen eine tagesstationäre Abrechnung der betrachteten Fälle hat. Zusätzlich wird betrachtet, inwiefern eine Prozessanpassung auf eine Entlassung aus dem AWR nach Mobilisierung Auswirkungen zeigt. Demgegenüber stehen die gleichen Betrachtungen ohne Einsatz eines Nahtverschlusssystems. In unserem Modell wurde der AWR dabei bewusst *Workflow-agnostisch* betrachtet, d. h. nur die Kapazität betrachtet, ohne zu unterscheiden, ob es sich um einen 24 h gepoolten AWR oder einen zweckgebundenen AWR z. B. für das Katheterlabor handelt.

### Validierung des Modells

**Abb. 2:**
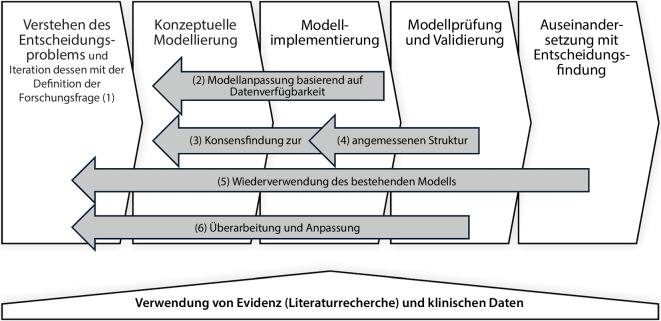
Entwicklungsprozess eines Ökonomie-Modells. (Adaptiert nach [23])

Um die Plausibilität und Robustheit des entwickelten Modells sicherzustellen, wurden mehrere iterative Validierungsmaßnahmen durchgeführt (Abb. 2). Die Annahmen und Berechnungsgrundlagen des Modells wurden mit Kardiolog:innen, Pflegekräften und Krankenhausökonom:innen diskutiert. Um die Robustheit des Modells zu testen, wurden Sensitivitätsanalysen durchgeführt. Zur zusätzlichen Absicherung der Modellgüte wurde eine Cross-Validierung durchgeführt, indem die Simulationsergebnisse durch alternative Rechenwege, in Abgleich mit unabhängigen externen Krankenhausberichten und gesundheitsökonomischen Analysen gebracht wurden. Dieser externe Abgleich trägt dazu bei, mögliche Verzerrungen durch die Verwendung der gleichen Datensätze für Modellannahmen und Evaluation zu minimieren.

## Ergebnisse

Die Arbeit analysiert in einer kombinierten Simulations- und Umsetzungsstudie die Auswirkungen des Einsatzes von Nahtverschlusssystemen auf klinische und ökonomische Parameter. Hierbei wurden 5 Szenarien modelliert, die sich hinsichtlich Verschlusstechnik, Abrechnungsart und Entlassungszeitpunkt unterscheiden (Tab. 2). Dabei kann eine effizientere Nutzung personeller und infrastruktureller Ressourcen sowohl die Fallzahlen im Grenzkostenbereich steigern als auch das Pflege- und Überwachungspersonal entlasten. Eine Reduktion des Personaleinsatzes wurde aufgrund gesetzlicher Personaluntergrenzen (PpUGV) sowie strukturellen Personalmangels, der bereits bestehende Prozesse limitiert [20], bewusst ausgeschlossen. Als Zielparameter wurden daher die Fallzahlsteigerung, die Reduktion der Personalbelastung, die Veränderung des Deckungsbeitrags sowie die gesundheitsökonomischen Effekte, gemessen in QALYs, identifiziert. Als Ausgangsszenario dient der Zustand ohne Einsatz eines Nahtverschlusssystems und ohne Prozess- und Abrechnungsumstellung. Die Ergebnisse der Sensitivitätsanalysen zeigten, dass das Modell stabil bleibt und auch bei konservativen Annahmen weiterhin positive Effekte durch die Einführung des Nahtverschlusssystems prognostiziert.

**Tab. 2:** Ausgabeparameter Simulationsmodell

Szenario	Zusätzliche Fälle pro Jahr (*n* = 1)	Deckungsbeitrag (in €)	Entlastung Personal (in %)	QALY-Gewinn
*Basis*	0	–	0	0,0
Nahtverschlusssystem ( )				
Tagesstationär ( )				
Prozessanpassung ( )				
*3.1*	0,065	3,00	7	0,0034
Nahtverschlusssystem (√)				
Tagesstationär ( )				
Prozessanpassung ( )				
*3.2*	0,065	415,00	7	0,0034
Nahtverschlusssystem (√)				
Tagesstationär (√)				
Prozessanpassung ( )				
*3.3*	0	388,80	0	0,0
Nahtverschlusssystem ( )				
Tagesstationär (√)				
Prozessanpassung ( )				
*3.4*	0,132	572,22	7	0,0
Nahtverschlusssystem ( )				
Tagesstationär (√)				
Prozessanpassung (√)				
*3.5*	0,25	661,27	24	0,004
Nahtverschlusssystem (√)				
Tagesstationär (√)				
Prozessanpassung (√)				

### Nahtverschlusssystem ohne Abrechnungs- oder Prozessanpassung

Der Einsatz eines Nahtverschlusssystems führte trotz unveränderter Prozesse und Abrechnung zu positiven Effekten: Die verkürzte Entlassungszeit – hier gemessen an der Aufnahme- und Entlasszeit im AWR – ermöglichte eine Fallzahlsteigerung von 6,5 % im Grenzkostenbereich und einen zusätzlichen Deckungsbeitrag von 3,00 € pro Fall – somit ergibt sich eine Kostenneutralität. Die Personalauslastung konnte um 7 % gesenkt werden, während der QALY-Zugewinn 0,0034 pro Patient:in betrug, entsprechend 3,4 QALYs bei 1000 Fällen.

### Nahtverschlusssystem mit tagesstationärer Abrechnung, ohne Prozessanpassung

Die Kombination aus Nahtverschlusssystem und tagesstationärer Abrechnung konnte den wirtschaftlichen Nutzen weiter steigern. Der Deckungsbeitrag stieg auf 415,00 € pro Fall. Aufgrund unveränderter Patientendurchlaufzeiten blieb die Personalentlastung bei 7 %. Das Szenario zeigt, dass durch die Abrechnungsanpassung in Kombination mit einem Nahtverschlusssystem eine messbare wirtschaftliche Optimierung erreicht werden kann. Der gesundheitsökonomische Nutzen blieb durch die weiterhin reduzierte Blutungswahrscheinlichkeit bei 9,5 % bestehen.

### Tagesstationäre Abrechnung ohne Nahtverschlusssystem oder Prozessanpassung

Allein durch die Umstellung auf die tagesstationäre Abrechnung ergab sich ein Mehrertrag von 388,80 € pro Fall. Da weder Fallzahl noch Komplikationsrate beeinflusst wurden, blieb ein QALY-Zugewinn aus. Die Ergebnisse zeigen wirtschaftliche Vorteile durch Abrechnungsanpassung, jedoch keinen Beitrag zur Versorgungsqualität.

### Tagesstationäre Abrechnung mit Prozessanpassung, ohne Nahtverschlusssystem

Dieses Szenario führte zu einer Fallzahlsteigerung um 0,132 pro Jahr, einem Mehrertrag von 572,22 € pro Fall und einer Personalentlastung von 7 %. QALY-Gewinne blieben aus, da keine Reduktion der Blutungskomplikationen vorliegt.

### Nahtverschlusssystem, tagesstationäre Abrechnung und Prozessanpassung

Das größte Effizienzpotenzial zeigte sich in der Kombination aus Nahtverschlusssystem, tagesstationärer Abrechnung und angepasster Prozessstruktur. Um unrealistische Verzerrungen zu vermeiden, wurde die Fallzahlsteigerung auf 25 % begrenzt, da laut Literatur und Experteninterviews [21] jenseits dieser Schwelle strukturelle Engpässe – etwa im Katheterlabor oder Personalbereich – zu limitierenden Faktoren werden. Innerhalb dieses Rahmens konnte die Verweildauer im AWR deutlich reduziert werden, was die Bettenverfügbarkeit und Behandlungskapazität verbesserte – rechnerisch 0,25 zusätzliche Fälle pro Basisfall bzw. 250 zusätzliche Fälle bei 1000 Fällen jährlich. Der Deckungsbeitrag stieg auf 661,27 € pro Fall, entsprechend 661.270 € bei 1000 Fällen. Gleichzeitig sank die Personalauslastung im AWR um 24 %, was eine spürbare Entlastung ermöglichte. Auch gesundheitsökonomisch zeigte sich dieses Szenario vorteilhaft: Der QALY-Zugewinn von 0,0034 pro Patient:in resultiert aus der reduzierten Komplikationsrate. Insgesamt stellt dieses Szenario die effektivste Strategie zur Maximierung klinischer und ökonomischer Effekte dar.

## Diskussion

### Zusammenfassung der Hauptergebnisse

**Abb. 3:**
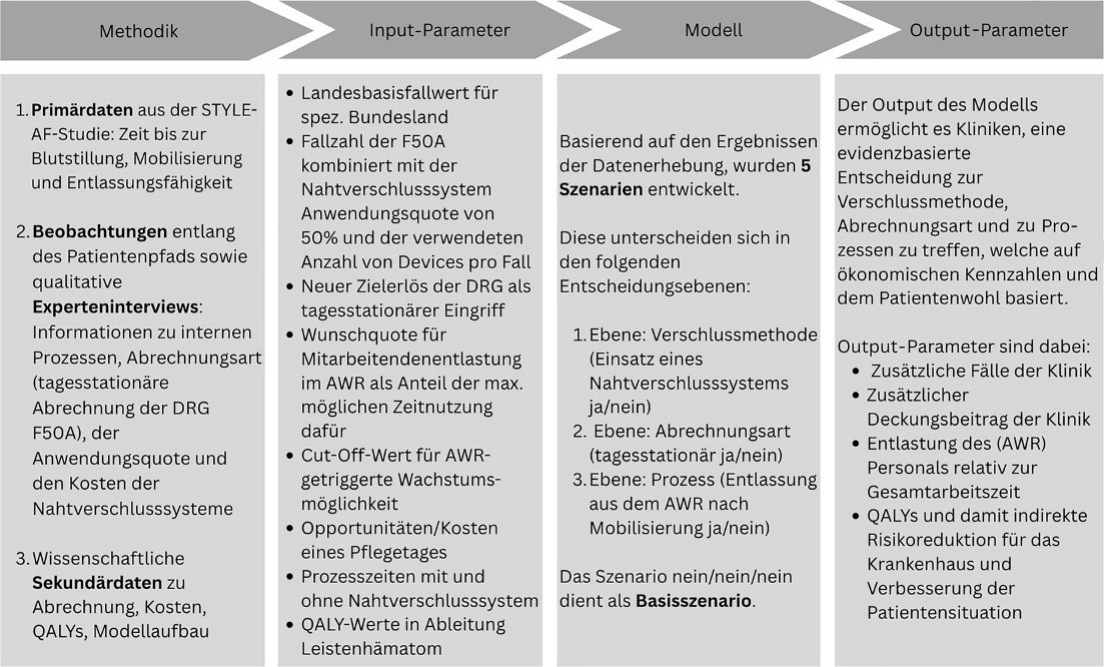
Strukturierte Vorgehensweise beim Modellaufbau

Die STYLE-AF-Studie zeigte, dass der Einsatz eines femoralen Nahtverschlusssystems (ProStyle) nach Katheterablation des Vorhofflimmerns die unmittelbaren postprozeduralen Abläufe deutlich verbessert, ohne Kompromisse bei der Sicherheit. So ließen sich allein durch den Einsatz des Verschlusssystems eine Fallzahlsteigerung um 6,5 % und ein QALY-Gewinn von 0,0034 pro Patient:in erreichen. In Verbindung mit der Umstellung auf tagesstationäre Abrechnung ergab sich eine deutliche Steigerung des Deckungsbeitrags (ca. +415 € pro Fall). Die größten Effekte zeigten sich im kombinierten Szenario (Nahtverschluss + frühe AWR-Entlassung + tagesstationär): Hier wurde eine Fallzahlsteigerung von 25 % erzielt, der Deckungsbeitrag stieg um 661 €/Patient:in, die Auslastung im AWR sank um bis zu 24 % und es resultierte erneut ein QALY-Zugewinn von 0,0034. Insgesamt weisen die Ergebnisse somit darauf hin, dass Nahtverschlusssysteme – insbesondere bei umfassender Implementierung ins Versorgungskonzept – gleichzeitig die klinischen Ergebnisse, die Prozesseffizienz und die Wirtschaftlichkeit verbessern können. Abb. 3 zeigt die strukturierte Vorgehensweise.

### Klinischer Nutzen und Sicherheitsprofil von Nahtverschlusssystemen

Unsere Ergebnisse bestätigen den klinischen Nutzen von Nahtverschlusssystemen im interventionellen Elektrophysiologie-Setting und untermauern deren Sicherheitsprofil. Diese Befunde stehen im Einklang mit aktuellen Studien: So zeigte eine multizentrische Propensity-Score-Analyse von 28.872 Katheterablationen [22], dass der routinemäßige Einsatz femoraler Verschlusssysteme die Gesamtkomplikationen signifikant reduzieren kann. Insbesondere vaskuläre Zugangskomplikationen und Blutungen traten in der Verschluss-Kohorte deutlich seltener auf (z. B. 0,83 % vs. 1,26 % für Gefäßkomplikationen in den ersten 7 Tagen; OR 0,66). Die Schlussfolgerung der Autoren lautet entsprechend, dass Nahtverschlusssysteme mit einer geringeren Komplikationsrate assoziiert sind als die manuelle Kompression. Neben der Sicherheit bietet der Verschluss auch direkte klinische Vorteile für die Patient:innen. Durch die mechanische Gefäßnaht wird eine sofortige Hämostase erreicht, was die Liegezeit unter Kompression oder Druckverband drastisch verkürzt. In der STYLE-AF-Studie resultierte dies in einer hochsignifikanten Verkürzung der Zeit bis zur Mobilisation (Time-to-Ambulation) im Mittel um mehrere Stunden. Ähnliche Ergebnisse wurden im AMBULATE-Trial [23] erzielt, welcher ein kardiologisches Verschlusssystem (VASCADE) gegen manuelle Kompression verglich. Bemerkenswert ist auch der Einfluss auf subjektive Outcomes: Sowohl STYLE-AF als auch AMBULATE berichten über eine höhere Zufriedenheit und geringeren Analgetikabedarf im Verschlussarm, was auf einen verbesserten Komfort und weniger Schmerzen an der Punktionsstelle hinweist. Diese patientenbezogenen Verbesserungen schlagen sich zusammen mit den beobachteten Änderungen im Bereich der Leistenblutung zwar nur geringfügig in QALY-Gewinnen nieder, sind aber klinisch relevant im Sinne einer schonenderen Behandlung. Mehrere aktuelle Untersuchungen adressieren auch alternative Hämostase-Methoden wie die Figure-of-Eight-Naht (kurze Umschlingungsnaht) als kostengünstige Option. Ein RCT-Vergleich zwischen einer Naht und dem ProGlide-Nahtsystem [24] fand nach großen venösen Zugängen keine signifikanten Unterschiede in der Komplikationsrate oder Zeit bis zur Hämostase. Zusammengefasst ergibt sich ein Bild, in dem Nahtverschlusssysteme den klinischen Workflow post Ablation erheblich erleichtern, ohne das Risiko für Patient:innen zu erhöhen. Im Gegenteil spricht die Evidenz für eine Reduktion von Gefäßkomplikationen und eine Verbesserung von Patientenerlebnis und -komfort durch den Verschlusseinsatz.

### Ökonomische Auswirkungen und QALY-Bewertungen

Die ökonomischen Analysen unserer Studie zeigen, dass der Einsatz von Nahtverschlusssystemen im Rahmen von AF-Ablationen in Abhängigkeit vom Abrechnungssystem zumindest kostenneutral, oft sogar finanziell vorteilhaft gestaltet werden kann. Der Deckungsbeitrag erhöht sich am meisten, wenn neben dem Verschluss auch prozessuale Änderungen wie die sehr frühe AWR-Entlassung umgesetzt werden. Wichtig ist zu betonen, dass diese Berechnungen den Krankenhausperspektiven entsprechen – so muss z. B. für eine Fallzahlerhöhung auch der Engpass im AWR liegen und eine Fallwarteliste vorliegen. Unsere Simulationen zeigen damit, dass Nahtverschlüsse bei optimaler Nutzung zu mindestens kostenneutralen Ergebnissen führen, bei voller Umsetzung sogar zu deutlicher Steigerung der Wirtschaftlichkeit und Effizienz. Ein weiterer Aspekt ist die gesundheitsökonomische Bewertung in Form von QALYs (Quality-Adjusted Life Years). Unsere Studie hat einen kleinen, aber messbaren QALY-Gewinn durch den Verschlusseinsatz ermittelt (≈0,0034 QALY pro Patient, basierend auf vermiedenen Blutungskomplikationen und erhöhtem Komfort). Dieser Wert erscheint auf den ersten Blick gering – er entspricht etwa 1,2 zusätzlichen gesunden Lebenstagen pro Patient –, summiert sich jedoch über große Patientenkohorten. Bei 1000 Ablationen mit Verschlusssystem entspräche dies z. B. rund 3,4 gewonnenen QALYs im Kollektiv. Ein solcher Zugewinn ist durchaus relevant, insbesondere da er mit praktisch keinen Mehrkosten einhergeht (oder sogar Kosteneinsparungen beinhaltet bei tagesstationärer Umsetzung). Nicht zuletzt unterstreichen aktuelle klinische Studien den patientenbezogenen Nutzen: Patienten mit Gefäßverschluss berichten eine bessere postprozedurale Lebensqualität und weniger Beschwerden [25] – Faktoren, die in traditionellen QALY-Berechnungen nur unvollständig abgebildet, aber für eine ganzheitliche Bewertung der Therapie wichtig sind.

Trotz der insgesamt positiven ökonomischen Perspektive dürfen mögliche Hürden nicht unerwähnt bleiben. Die Einführung von Nahtverschlusssystemen erfordert initial Schulungen und Anschaffungen. Zudem fallen pro Eingriff Materialkosten an, die je nach Land nicht spezifisch erstattet werden. Unsere Simulation geht von einer gleichbleibenden personellen Besetzung aus – eine Reduktion von Personal durch Effizienzgewinne haben wir bewusst nicht eingerechnet. Unsere Ergebnisse liefern jedoch evidenzbasierte Hinweise, dass unter günstigen Rahmenbedingungen sowohl aus klinischer als auch ökonomischer Sicht kein Nachteil durch den Verschlusseinsatz entsteht – im Gegenteil, es zeigen sich Chancen für Qualitäts- und Erlössteigerungen.

### Workflow-Optimierung und Krankenhauslogistik

Die Implementierung von Nahtverschlusssystemen entfaltet ihre größten Effekte im Zusammenspiel mit optimierten Prozessen und einer darauf abgestimmten Krankenhauslogistik. Ein zentrales Ergebnis unserer Untersuchung ist die erhebliche Reduktion der Verweilzeit im AWR nach Ablation bei Verwendung des Verschlusssystems. In unserem Simulationsmodell wurde explizit der Effekt einer frühen AWR-Entlassung analysiert: Er führte – abhängig von vorhandenen Kapazitätsgrenzen – zu einer Entlastung des AWR-Personals um bis zu 24 % und trug maßgeblich zur genannten Fallzahlerhöhung bei. Praktisch bedeutet dies, dass pro Tag mehr Ablationspatient:innen versorgt werden können, da Betten und Monitorressourcen im AWR schneller wieder verfügbar sind. Dieser Befund ist hochrelevant, weil in vielen Elektrophysiologie-Zentren der AWR ein engpasskritischer Faktor ist. Darüber hinaus erleichtert der sichere Gefäßverschluss die tagesgleiche Entlassung bzw. Beurlaubung über Nacht von Patient:innen. Dies setzt natürlich ein angepasstes organisatorisches Workflow-Management voraus: Früh am Tag beginnende Eingriffe, strukturierte Entlassungsuntersuchungen am Nachmittag, und eine enge Nachverfolgung am Folgetag. Unsere Daten liefern hierfür eine rationale Grundlage, indem sie quantifizieren, wie stark die Prozesszeiten verkürzt werden können. Unsere Simulation bestätigt dies und demonstriert in praxi, dass die Kombination aus Verschlusssystem und Umstellung der Prozesse wesentlich zur Effizienzsteigerung beiträgt. Auch qualitativ ergeben sich Vorteile im Workflow: Patienten können abends in vertrauter Umgebung sein, und das Pflegepersonal wird von Routineüberwachungsaufgaben in den Nachtstunden entlastet. Indem außerdem Überwachungsbetten und Normalstationskapazitäten eingespart oder schneller wieder frei werden, schafft man Luft für andere Patienten. Letztlich bedeutet jede vermiedene Übernachtung eine Opportunität, entweder zusätzliche Fälle zu behandeln oder Ressourcen (Personal, Betten) einzusparen. In Zeiten limitierter Gesundheitsressourcen und Pflegepersonalknappheit ist dies ein wichtiger strategischer Vorteil.

Auch zeigt sich in unserem flexiblen Modell, dass die Engpässe je nach Klinik variieren können (AWR vs. Katheterlabor vs. Bettenkapazität) – durch flexible Anpassung der Prozesse lässt sich aber identifizieren, wo der Verschlusseinsatz den größten Hebel hat. So könnten Krankenhäuser mit hoher Auslastung im AWR besonders profitieren, während in anderen Einrichtungen das Katheterlabor der begrenzende Faktor bleibt. In letzterem Fall müsste z. B. geprüft werden, ob die verkürzte AWR-Zeit tatsächlich mehr Prozeduren pro Tag ermöglicht oder ob das Personal im Katheterlabor die eigentliche Engstelle darstellt. Insgesamt bietet der Einsatz von Nahtverschlusssystemen Kliniken eine wertvolle Option zur Workflow-Optimierung: Er reduziert Wartezeiten, entlastet die Pflege und steigert die Versorgungskapazität, was in Summe die Effizienz der Rhythmologie-Abteilung erhöht.

### Limitationen und Generalisierbarkeit

Trotz der vielfältigen positiven Implikationen müssen die Limitationen unserer Studie und die Übertragbarkeit der Ergebnisse kritisch beleuchtet werden. Erstens basiert die ökonomische Analyse auf einem simulationsbasierten Modell, das auf Daten eines einzelnen Zentrums (STYLE-AF-Studie) und Expertenannahmen fußt. Auch wenn umfangreiche Validierungen und Sensitivitätsanalysen durchgeführt wurden, bleibt eine inhärente Unsicherheit bestehen. Andere Krankenhäuser könnten abweichende Prozesszeiten, Kostenstrukturen oder Komplikationsraten aufweisen, was die absoluten Effekte beeinflusst. Die prospektive STYLE-AF-Studie war so auch mit 125 Patient:innen relativ moderat in der Fallzahl, sodass seltene Komplikationen oder Langzeitergebnisse nicht erfasst werden konnten. Obwohl keine Major-Komplikationen im Verschlussarm auftraten, ist die statistische Power begrenzt. Eine groß angelegte multizentrische Studie wäre wünschenswert, um die Sicherheitsdaten zu untermauern – insbesondere im Hinblick auf sehr seltene Ereignisse wie Gefäßverschlussstörungen, Infektionen an der Punktionsstelle oder Device-bezogene Probleme. Bisherige Register [26] geben hier aber Entwarnung und sehen keine Häufung solcher Komplikationen. Weiterhin ist die Generalisierbarkeit der ökonomischen Ergebnisse eingeschränkt. Unser Krankenhaus war ein tertiäres Zentrum mit hoher Ablationsfrequenz und bestehenden Strukturen. Kleine Häuser mit geringerer Ablationszahl könnten die in unserem Modell angenommene Steigerung der Fallzahlen (bis +25 %) nicht realisieren, da sie möglicherweise nicht über genügend Nachfrage oder Personal verfügen. Ebenso setzen unsere Workflow-Verbesserungen voraus, dass zu jedem Zeitpunkt genug Patient:innen vorhanden sind, um die freiwerdenden Kapazitäten zu nutzen – was in der Praxis durch Terminplanung und Patientenselektion begrenzt sein kann. Schließlich ist darauf hinzuweisen, dass unser QALY-Modell nur ausgewählte Endpunkte einbezieht (v. a. Vermeidung von Blutungskomplikationen und verkürzte Liegezeiten). Langzeit-QOL-Daten (z. B. Lebensqualität Wochen nach dem Eingriff, Chronifizierung von Leistenschmerzen etc.) liegen bislang kaum vor. Mögliche langfristige Vorteile – etwa weniger chronische Beschwerden durch Hämatome – könnten die QALY-Bilanz noch verbessern, wurden aber mangels Daten konservativ unberücksichtigt gelassen. Umgekehrt haben wir keine potenziellen negativen Langzeitfolgen (wie Narbenprobleme oder Gefäßstenosen am Punktionsort) in die Rechnung aufgenommen, da hierzu keine Evidenz besteht.

Trotz dieser Einschränkungen glauben wir, dass die Kernaussagen unserer Studie robust sind. Die Resultate wurden durch Sensitivitätsanalysen auf Plausibilität geprüft und blieben auch unter veränderten Annahmen wie z. B. Kosten der Systeme oder 2 Devices pro Eingriff auch weit über dem Listenpreis o. Ä. im Grundsatz valide (d. h. der Verschlusssystem-Einsatz zeigte konsistent neutrale bis positive Effekte). Nichtsdestotrotz sollten zukünftige Untersuchungen in unterschiedlichen Versorgungssettings prüfen, inwieweit unsere Ergebnisse replizierbar sind. Die Limitationen unterstreichen, dass es kein *One-size-fits-all*-Szenario gibt – die Implementierung von Nahtverschlusssystemen muss immer im Kontext der jeweiligen Klinik betrachtet werden, inklusive Personalstruktur, Budgetierung und Patientenkollektiv.

### Ausblick: Zukünftige Forschung und klinische Umsetzung

Die vorliegenden Erkenntnisse eröffnen mehrere Perspektiven für die zukünftige Entwicklung in der interventionellen Rhythmologie. Ein offensichtlicher nächster Schritt ist die Durchführung größerer multizentrischer Studien, die die Vorteile von Nahtverschlusssystemen weiter quantifizieren.

Ein weiterer Forschungsbereich betrifft die Patientenselektion und Protokolloptimierung. Zwar ist bekannt, dass eine tagesstationäre bzw. Same-day-discharge-Behandlung für einen Großteil der AF-Ablationspatient:innen machbar ist, doch fehlen klare Kriterien, welche Patient:innen zwingend überwacht werden sollten (z. B. hohes Blutungsrisiko, weite Anreise, fehlende Betreuung daheim etc.). Zukünftige Arbeiten könnten Risikoscoring-Modelle entwickeln, um diejenigen Fälle zu identifizieren, die auch mit Verschluss besser stationär aufgenommen bleiben.

Weiter spielen im Prozess auch strukturelle Voraussetzungen eine Rolle – etwa die Verfügbarkeit einer 24/7-Hotline oder einer Notfallaufnahme, falls es nach Entlassung zu Komplikationen kommt. Die Implementierungsforschung könnte untersuchen, wie Kliniken verschiedenster Größe die Prozesse und Pfade erfolgreich etablieren können (Change-Management, Schulungen, Patientenaufklärung). Unsere Ergebnisse legen nahe, dass die Bereitschaft zur Prozessanpassung ein Schlüsselfaktor ist, um den vollen Nutzen der Verschlusstechnologie auszuschöpfen. Auf gesundheitspolitischer Ebene könnte der Nachweis des Nutzens von Nahtverschlusssystemen dazu führen, dass diese Technologie vermehrt in Qualitätsinitiativen und Leitlinienempfehlungen berücksichtigt wird. Denkbar wären Zertifizierungskriterien für EP-Zentren, die u. a. einen Anteil an SDD-Ablationen oder den Einsatz moderner Gefäßmanagement-Methoden umfassen. Ebenso könnten Kostenträger bei klar belegter Kosteneffektivität erwägen, Pay-for-Performance-Elemente einzuführen – etwa Boni für Kliniken, die geringe Komplikationsraten und kurze Liegezeiten bei AF-Ablation vorweisen (analog zu bestehenden Qualitätsverträgen). Hierfür ist jedoch zunächst weitere Evidenz nötig, um Entscheidungsträger zu überzeugen.

Nicht zuletzt regt unsere Studie Überlegungen an, wie die gewonnenen Ressourcenfreiheiten genutzt werden können. Die Tatsache, dass mit gleicher Infrastruktur mehr Patient:innen behandelt werden können, ist im Angesicht steigender Prävalenzen ein willkommenes Ergebnis. Künftig könnte dies dazu beitragen, Wartelisten für Ablationen zu verkürzen und dem erwartbaren Anstieg an Ablationsindikationen (auch durch Ausweitung der Indikationen in Leitlinien) besser gerecht zu werden. Allerdings muss parallel die Personalfrage gelöst werden – Effizienzgewinne entbinden nicht von der Notwendigkeit, ausreichend Fachpersonal auszubilden und vorzuhalten. Hier könnte perspektivisch die Entlastung des Pflegepersonals durch Verschlusssysteme zu einer höheren Attraktivität der AWR-/Stationsarbeit führen, was die Bindung von Pflegekräften unterstützt – ein Aspekt, der in künftigen Untersuchungen z. B. mittels Arbeitsbelastungsscores evaluiert werden könnte.

Zusammenfassend bieten Nahtverschlusssysteme einen vielversprechenden Ansatz, um den steigenden Anforderungen in der interventionellen Rhythmologie zu begegnen. Zukünftige Forschung wird darauf abzielen, die Balance zwischen Aufwand und Nutzen weiter zu verfeinern: Wie lassen sich maximale klinische Vorteile (höhere Sicherheit, Zufriedenheit) und ökonomische Vorteile (geringere Kosten, höhere Effizienz) erzielen, ohne neue Risiken einzuführen? Unsere Studie liefert hierfür einen Grundstein. Die nächsten Jahre werden zeigen, ob sich diese Technik flächendeckend durchsetzt und möglicherweise zum neuen Standard in der Ablationsnachsorge wird – zum Wohle der Patient:innen und der Gesundheitssysteme.

## Data Availability

Das Material, das in die Rechnungen zu dieser Studie eingeflossen ist, ist öffentlich verfügbar. Diese liegen über die Style-AF-Studie und aggregierte Zahlen z. B. bei Personalkosten per InEK-Schema und anderen publizierten Quellen vor. Weitere genutzte Daten werden nicht öffentlich zur Verfügung gestellt.

## Footnotes

1 DRG F50A: Ablative Maßnahmen bei Herzrhythmusstörungen mit hochkomplexer Ablation im linken Vorhof, Ventrikel oder Pulmonalvenen oder Implantation eines Ereignisrekorders.
